# The Neurocognitive Study for the Aging: Longitudinal Analysis on the Contribution of Sex, Age, Education and *APOE* ɛ4 on Cognitive Performance

**DOI:** 10.3389/fgene.2021.680531

**Published:** 2021-07-13

**Authors:** Andreas Chadjikyprianou, Marilena Hadjivassiliou, Savvas Papacostas, Fofi Constantinidou

**Affiliations:** ^1^Department of Psychology and Center for Applied Neuroscience, University of Cyprus, Nicosia, Cyprus; ^2^Department of Cardiovascular Genetics and Laboratory of Forensic Genetics, The Cyprus Institute of Neurology and Genetics, Nicosia, Cyprus; ^3^Cyprus Institute of Neurology and Genetics, The University of Nicosia Medical School, Nicosia, Cyprus

**Keywords:** cognitive aging, aging, episodic memory, executive functions, cognitive trajectories, cognitive stability, STROBE, cohort

## Abstract

**Objective:** The effects of normal cognitive aging on executive functions (EF), Verbal Episodic Memory (VEM) and the contribution of age, sex, education, and APOΕ ε4 in a group of old Greek Cypriots across a five-year period were investigated.

**Design:** NEUROAGE, the first project on cognitive aging in Cyprus, is a prospective longitudinal study with a rolling admission process. Participants are assessed at baseline and retested every 24–30 months.

**Subjects:** 170 participants completed all three testing cycles; 86 men and 84 women with ages ranging between 60 and 88 years (mean = 73.21, *SD* = 5.84); education, 2–20 years (mean = 9.07, *SD* = 4.27).

**Results:** Α Repeated Measures Multivariate Analysis of Covariance was conducted with one between-subject factor: sex; two covariates: age and education, while Time (time 1, time 2, time 3) served as a within – subject factor. Time did not have an effect on mini mental status examination in Greek (MMSE), EF or VEM. Also, sex had no effect on MMSE, EF and VEM. There was no time by sex interaction. Age and Education significantly predicted the EF performance, *F*(1, 168) = 11.23, *p* < 0.05; *F*(1, 158) = 90.03, *p* < 0.001 and VEM performance, *F*(1, 171) = 17.22, *p* < 0.001; *F*(1, 171) = 61.25, *p* < 0.001. Furthermore, there was a significant interaction effect between time and education, for EF, *F*(2, 167) = 7.02, *p* < 0.001. Performance of the *APOE* ε4 carriers did not differ on any of the above measures as compared to performance of non-carriers in this older adult group.

**Conclusion:** Cognitively healthy adults maintained overall cognitive performance across the five-year period. Male and female participants performed similarly and the pattern of change over time was similar across the two sexes. Education was predictive of VEM and EF performance across time. Furthermore, those with higher education maintained higher levels of EF performance. *APOE* results did not differentiate performance at baseline. Implications of findings are discussed.

## Introduction

The 2017 global action plan on the public health response to dementia, estimates that 47 million people worldwide have dementia and the prevalence is expected to rise due to increases in life expectancy (World Health Organization, 2017). A significant number of studies have been focusing on the pathogenesis of dementia and potential protective factors. The present study is part of a systematic effort to describe and characterize cognitive changes associated with healthy aging using a longitudinal design.

Research indicates that the trajectory of cognitive change varies for different cognitive functions (Schaie, 2005). Changes in cognitive performance associated with aging are probably a result of neurobiological processes that occur during the lifespan. Prefrontal cortical areas, inferior temporal lobe areas and the hippocampus sustain the greatest diminution of neuronal loss, blood flow, and shrinkage (Kramer et al., 2007; Rajah et al., 2010). While some studies show a decline in cognitive functions such as episodic memory (Lundervold et al., 2014), verbal fluency (Kosmidis et al., 2004) and mental flexibility (Wecker et al., 2005; Thomas and Kunzmann, 2014), with increasing age, other studies report an age-related advantage in cognitive abilities such as general and semantic knowledge (Hedden and Gabrieli, 2004). The latter, rely on knowledge and information acquired over time to form the theoretical construct of crystallized intelligence which is resistive to the clinical manifestations of cognitive aging (Giogkaraki et al., 2013).

The present study is part of our systematic research program on the Neurocognitive Study for the Aging (NEUROAGE). NEUROAGE is the first longitudinal study on cognitive aging in Cyprus. It explores modifiable and unmodifiable factors that contribute to cognitive health in the Greek-Cypriot population of Cyprus with its unique social-cultural, linguistic and genetic characteristics (Constantinidou et al., 2012a, 2015; Giogkaraki et al., 2013; Demetriou and Constantinidou, 2018). The present study is the first report on the longitudinal data from the NEUROAGE study.

Specifically, we investigated the stability of performance across a five-year period on two neuropsychological domains – verbal episodic memory (VEM), and executive functions (EF), two-well studied cognitive domains known to be affected by cognitive aging (Lezak, 2012). Most importantly, we explored the potential contribution of important demographic and genetic factors previously reported in the literature, namely sex, age, education, and *APOE* in cognitive performance (Constantinidou et al., 2014; Caselli et al., 2015).

VEM and EF are not completely independent processes. Studies with cognitively healthy individuals indicate that deficits in EF have an indirect impact on memory performance (Spaan, 2015). Theoretical models of working memory (WM) posit a central executive, or a common attentional control mechanism similar to the central executive mode of WM proposed by Baddeley (2000). Executive functions could be viewed as a supervisory system that is involved in the coordination and control of goal-directed behavior, including WM (Constantinidou et al., 2012b; Constantinidou and Thomas, 2017).

Some studies provide evidence that VEM declines in a linear fashion as a function of age (Park et al., 2002; Lundervold et al., 2014) starting at the age of 30 (Constantinidou et al., 2014). In contrast, others suggest a later start, between 65 and 70 years (Rönnlund et al., 2005). Methodological factors such as sample size and statistical power issues, longitudinal vs. cross-sectional designs, and complexity of WM tasks may in part account for these differences.

EF is a multidimensional construct incorporating planning/initiation, execution, self-regulation/monitoring and effective performance (Lezak, 2012). EF activities dependent on speed of processing and strategic planning are more sensitive to aging (Treitz et al., 2007). Age-related changes in EF can have a direct impact on memory performance because of their important role in implementation of encoding and retrieval strategies (Constantinidou et al., 2012b; Lezak, 2012).

In addition to age, the present study explored the contribution of education to cognitive aging across time. Specifically, existing research suggests that years of formal education independently affect cognitive performance in both crystallized (i.e., vocabulary and semantic knowledge) and fluid intelligence tasks requiring online processing (i.e., visual construction, verbal fluency and WM; Meguro et al., 2001; Bosma et al., 2003; Stern, 2006). Higher educational level mitigates cognitive decline in older adults without dementia because it promotes efficient cognitive processing and better cognitive performance (Bosma et al., 2003).

While higher education may result in improved cognitive performance in cross-sectional studies, it is not clear whether it safeguards cognitive stability over time. Some longitudinal studies found no evidence to support the notion that years of education moderate the decline of WM and EF (Zahonde et al., 2011) as measured by verbal learning, long-term memory, set shifting, and semantic fluency tasks (Van Dijk et al., 2008; Cadar et al., 2017). These results support the passive hypothesis of cognitive reserve in which older people with higher education continue to perform at a higher level than people with lower education; however, the slope of change in performance, is not influenced by education (Ritchie et al., 2016).

The divergence between the reports on the exact contribution of education to cognitive decline, may be due to methodological differences in study design, and lack of sensitivity of the cognitive tests implemented. The present study addresses the above limitations by incorporating tests with demonstrated sensitivity to cognitive aging and a longitudinal design with three time points in order to minimize measurement error (Winkens et al., 2006).

In addition to age and years of education, other unmodifiable factors such as sex and genetic risk were taken into consideration in order to study cognitive changes across time. Research has shown that women have an advantage in VEM as compared to men, whereas potential male advantage in visuospatial memory is not well delineated (De Frias et al., 2006; Karlsson et al., 2015). Previous research has provided mixed evidence for sex differences in EF (Munro et al., 2012; McCarrey et al., 2016; Zaninotto et al., 2018). How sex differences interact with cognitive aging remains unclear. The present study explores the contribution of sex as a biological construct in cognitive performance across time for both VEM and EF.

The APOΕ ε4 allele has been implicated as a primary genetic risk factor in pre-clinical and late-onset dementia and cognitive decline (Reas et al., 2019). The *APOE* gene expresses the protein apolipoprotein ε. There are three slightly different versions (alleles) of the *APOE* gene. The major alleles are called ε2, ε3, and ε4. The most common allele is ε3, which is found in more than half of the general population. Possession of the ε4 allele has been associated with poorer cognitive abilities and more rapid longitudinal decline in healthy older people, particularly in episodic memory (Shin et al., 2014; Marioni et al., 2016). The allele ε4 is considered a biological risk factor for cognitive decline.

Over the long history of life on this island, the Greek-Cypriot population has sustained several population effects that shaped the gene pool accordingly. Examples to this effect have been the many well documented founder effects pertaining to several diverse monogenic diseases, including neurological, endocrine and renal conditions (Pierides et al., 2009). Smaller or greater migration waves during the history of Cyprus as well as events relating to persecutions during wars, concerning a rather small population of just a few hundred thousands, favored genetic drifts, bottlenecks and founder phenomena, all having left behind their signs, either on the population island-wide, on selected geographic isolates, or even on selected religious minorities (i.e., Armenians, Maronites). The genetic characteristics of a relatively homogeneous population provide unique opportunities for health research. For example, the primary genetic risk factor for dementia, the APOΕ ε4 allele, is lower in Cyprus as compared to other European countries (Cariolou et al. 1995). Yet, dementia is the 7th leading cause of death and the primary cause of loss of health in Cypriots (World Health Organization, 2017).^1^

The present work implemented a prospective longitudinal design to examine the effects of normal cognitive aging on mental status, EF and VEM performance in a group of healthy old Greek Cypriots across a five-year period. The aim of the present study was also to examine sex differences in mental status and domain-specific cognitive performance (EF, VEM) in relation to both, levels of performance at baseline and across time. Finally, the present study aimed to determine the contribution of age, education and APOΕ ε4. The dependent measures implemented in this study were selected carefully in order to provide multiple data on VEM and EF, two neurocognitive domains that are sensitive to the effects of aging and brain pathology.

It was hypothesized that after in a five-year period, participants would present statistically significant signs of cognitive decline on VEM and EF. We also hypothesized that women would show an advantage in VEM performance, whereas no sex effects were expected for EF performance. Hypotheses regarding sex differences in patterns of change were less clear due to the lack of fully consistent findings in prior studies. We also hypothesized that age and education would influence the patterns of change across time. Finally, we hypothesized that the performance of individuals with the *APOE* ε4 allele would be significantly lower on VEM, EF and mini mental status examination in Greek (MMSE) as compared to non-carriers.

## Materials and Methods

The NEUROAGE began in 2009 and is the first longitudinal project of cognitive aging in Cyprus (clinicaltrials.gov Identifier: NCT01481246). With a prospective design and a rolling admission process, the study recruits Greek Cypriot volunteers from community settings in compliance with the Helsinki Declaration and following approval by the National Bioethics Committee (EEBK/ΕΠ/2008/26). Participants are assessed at baseline and are followed up every 2 years (follow up time range = 24–30 months). The average time of follow up for Time 3 was 57 months.

### Participants

**Figure 1:**
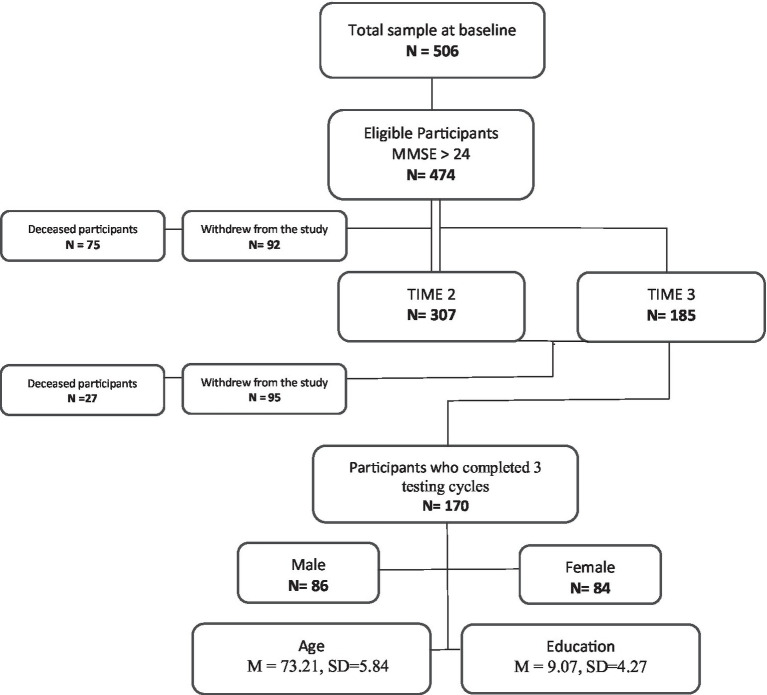
STROBE flow diagram.

Five hundred and six Greek Cypriot men and women were recruited from the NEUROAGE project who completed baseline evaluations. Thirty-two participants were excluded from the study due to a baseline diagnosis of MCI (*n* = 5), possible diagnosis of Alzheimer’s dementia (*n* = 4), and a Mini Mental Score Examination under 24, without a clinical diagnosis (*n* = 23). Out of the 474 who met study inclusion criteria, 307 participants were retained for the second evaluation (Time 2) at 24–30 months after their baseline assessment; 185 participants completed the third evaluation (Time 3) at 24–30 months after Time 2. Figure 1 is the STROBE diagram. A total of 170 study participants completed all three assessment cycles (Baseline, Time 2 and Time 3) and were retained in the analyses. Out of the 170 participants, 86 were men and 84 were women with ages ranging between 60 and 88 years (mean = 73.21, *SD* = 5.84). Education, 2–20 years (mean = 9.07, *SD* = 4.27). The demographic distribution (age, gender, and education) of the study was consistent with the Cyprus Government census data (Cyprus Statistical Service, 2011).

All participants were community dwellers residing independently at home. They were recruited from senior social clubs/centers, press-releases and outreach activities. The inclusion - exclusion criteria for all participants were the following: (1) Native Greek speakers, (2) males and females over the age of 60, (3) Good general health with no previous history of neurological disorder such as head trauma, stroke or neurodegenerative disorder, (4) No history of severe psychiatric or emotional disorder requiring hospitalization, (5) Baseline MMSE score of 24 or higher, and (6) Baseline Geriatric Depression Score of 6 or lower.

### Procedures

Participants were administered a battery of neurocognitive tests (translated and adapted into Greek and previously used in other research studies), sensitive to detect age-associated cognitive changes (Constantinidou et al., 2012a). Following are the measures included in the study.

### General Cognitive Screening

- Mini Mental Status Examination in Greek was used a quick cognitive screening (Fountoulakis et al., 2000). A score under 24 (out of possible 30) was the cutoff for study inclusion to exclude participants with dementia, taking into account individuals with lower education (Constantinidou et al., 2012a).

### Executive Functioning Tests

- Greek Version of the Trail Making Tests (TMT) A and B (Zalonis et al., 2008): The TMT provides information on visual search, scanning, graphomotor coordination, speed of processing, mental flexibility, and EF.
- Symbol Digits Modalities Test (Smith, 1982): It assesses complex scanning, visual tracking, and eye hand coordination within a time constraint. The total number of items correct in 90 s was used in the analysis.
- Greek Version of the Verbal Fluency. Two verbal fluency tasks: Animal recall and Words from the letter F were implemented, modified from the Controlled Oral Word Association Test (Kosmidis et al., 2004). The total correct items retrieved in 60 s for each test was used in the analyses.

### Verbal Episodic Memory

- Hopkins Verbal Learning Test–Revised (Greek version; HVLT; Benedict et al., 1998); adapted in Greek by Constantinidou upon permission by the publisher (Constantinidou et al., 2015; Philippou et al., 2018). Learning trials (first trial and the total score of the three trials), delayed recall and delayed recall performance scores were used.
- Greek version of the Logical Memory Story A and B adapted from the Wechsler Memory Scale – Revised: Immediate and delayed recall administrations of short story material (Wechsler, 1997; Constantinidou and Ioannou, 2008).

### *APOE* Genotyping

DNA was extracted from whole-blood samples from 308 study participants. The *APOE* molecular genotyping was carried out as described before in Cariolou et al. (1995). The genotypes of the ε2, ε3 and ε4 alleles were estimated by the gene counting method.

### Data Scoring and Analyses

The primary interest of the current study was to determine the effects of time, on MMSE, EF and VEM abilities. Repeated Measures multivariate analysis of covariance was conducted, to determine statistical difference in MMSE, EF and VEM performance across Time (time 1, time 2, time 3). Sex was entered as a between-subject factor. Years of education and age, were entered as covariates in a continuous format. For a small effect size (0.30) and three repeated measures, 39 participants are required for an *α* = 0.05 and power = 0.80 to detect significant effects. For the two group comparisons, 75 participants per group are required for an *a* = 0.05 and power = 0.80 to detect small group effects (0.25) and 35 participants per group for moderate group effects (0.60) across two variables (Stevens, 2009).

## Results

A principal component analysis (PCA) was run on the six memory and five EF tests in order to maximize reliability and generalizability, and minimize idiosyncratic aspects of single measures. The suitability of PCA was assessed prior to analysis. Inspection of the correlation matrix showed that all variables had at least one correlation coefficient greater than 0.3. The overall Kaiser-Meyer-Olkin measure was 0.89. Bartlett’s test of sphericity was statistically significant (*p* < 0.0001), indicating that the data was likely factorizable. Principal component analysis revealed two components that had eigenvalues greater than one, explaining 56.5 and 11.9% of the total variance, respectively. Visual inspection of the scree plot indicated that the two components can be retained. An oblimin rotation was employed to aid interpretability. As seen in Table 1, two components were extracted, the first component was labeled VEM and the second component EF.

**Table 1:** Factor loadings for each test to VEM and EF components.

	Verbal episodic memory	Executive functions
**Items**		
HVLT trial 1–3 total	**0.60**	−0.28
HVLT trial 4, Delayed recall	**0.66**	−0.21
Logical Memory – A, Immediate recall	**0.90**	0.07
Logical Memory – B, Immediate recall	**0.88**	0.04
Logical Memory – A, Delayed recall	**0.94**	0.06
Logical Memory – B, delayed recall	**0.85**	−0.01
Trail - A	0.08	**0.90**
Trail - B	0.00	**0.87**
Symbol digit modalities Test, correct	0.08	**−0.81**
Animals	0.32	**−0.47**
Words from F	0.00	**−0.63**

Following the PCA, composite scores were calculated for VEM and EF as they can be more powerful than their constituent parts for detecting change (Constantinidou et al., 2012a). Initially, scores were valenced so that a lower score indicated worst performance. Individual test scores obtained from each participant were transformed into z-scores (standard score) based on the mean of all the participants. Finally, the calculated standard scores from each test were averaged to derive a score for each construct.

### *APOE* Gene Distribution

Table 2 is the genotype and allele relative frequencies in the sample analyzed. The distribution of *APOE* genotypes in the present study population was as follows: 76.5% were homozygotes for the ε3 allele, 11.6% were heterozygotes for the ε2 and ε3 alleles, 0.6% were homozygotes for the ε2 allele, and 9.74% were heterozygotes for the ε3 and ε4 alleles. Thirty-seven participants (12%) had at least one ε4 allele. For purpose of analysis, we categorized participants as *APOE* ε4 allele carriers and non-carriers.

**Table 2:** Genotypic and allelic frequencies of *APOE* polymorphism in the sample analyzed (N308).

*APOE* genotypes	Number of individuals (relative frequency)
ε2 - ε2	2 (0.65)
ε2 - ε3	36 (11.69)
ε2 - ε4	5 (1.62)
ε3 - ε3	233 (75.65)
ε3 - ε4	30 (9.74)
ε4 - ε4	2 (0.65)
**Allele**	
ε2	45 (7.30)
ε3	532 (86.36)
ε4	39 (6.33)

The study population was in Hardy–Weinberg equilibrium (*X*^2^ = 2.836, 3df; *p* = 0.418).

### Time, Sex, Age, Education and Mini Mental State Examination

The first analysis examined the effect of Time on MMSE while sex was conducted as between-subject factors and age and education included as covariates. Preliminary checks were conducted to ensure that there was no violation of the assumptions of normality, linearity, homogeneity of variances, and reliable measurement of the covariate. Time did not yield significant changes on MMSE performance, *F*(2, 176) = 0.520, *p* = 0.59, partial *η*^2^ = 0.006, observed power = 0.13. None of the other main effects (sex; age; education) or multivariate interactions (time × age; time × education; time × sex) were significant. These finding indicate that participants exhibited no significant change on MMSE across the three testing periods and other important variables such as age, sex and education were not associated with changes on MMSE performance across time. The means and standard deviations displayed in Table 3, also indicate non-clinically significant changes across time for the individual tests and composites.

**Table 3:** Means and standard deviations on MMSE, Executive Functioning (EF) tasks and Verbal Memory tasks by Age and Educational Group by sex groups and time.

	Female			Male		
	TIME 1	TIME 2	TIME 3	TIME 1	TIME 2	TIME 3
MMSE	27.31 (1.81)	27.04 (2.22)	27.06 (1.95)	27.21 (1.74)	28.78 (2.75)	27.43 (1.70)
EF composite	0.288 (0.420)	0.299 (0.446)	0.229 (0.385)	0.297 (0.412)	0.281 (0.470)	0.205 (0.381)
TMT A	80.71 (35.54)	78.48 (29.46)	81.86 (53.59)	85.53 (44.43)	85.87 (29.15)	89.66 (45.16)
TMT B	181.46 (73.69)	188.51 (68.83)	198.14 (94.71)	191.66 (90.74)	192.51 (78.69)	203.99 (101.83)
SDMT	24.01 (7.76)	23.71 (7.72)	23.19 (7.75)	24.92 (7.46)	23.64 (7.72)	23.01 (7.75)
Animals	10.50 (2.67)	10.95 (3.18)	10.47 (3.12)	11.05 (2.96)	10.43 (3.11)	10.63 (3.49)
F Words	8.30 (3.06)	8.37 (3.23)	7.88 (3.32)	8.05 (3.31)	7.81 (3.29)	7.71 (3.04)
VEM composite	0.184 (0.648)	0.230 (0.691)	0.210 (0.738)	0.171 (0.667)	0.087 (0.656)	0.078 (0.623)
HVLT Trial 1	4.81 (1.58)	4.84 (1.59)	4.97 (1.37)	4.43 (1.66)	4.50 (1.73)	4.46 (1.77)
HVLT Trial 2	6.68 (1.81)	6.88 (1.93)	7.03 (1.34)	6.11 (1.81)	5.98 (1.93)	6.11 (1.97)
HVLT Trial 3	7.51 (2.10)	7.89 (1.98)	7.75 (2.24)	6.82 (2.06)	6.92 (2.23)	6.87 (2.10)
HVLT Trial 4	5.37 (2.84)	5.77 (2.56)	5.51 (2.72)	4.50 (2.82)	4.90 (2.91)	4.85 (2.62)
LMA	10.52 (3.57)	11.15 (3.82)	10.40 (4.16)	11.03 (4.17)	10.79 (3.79)	9.89 (3.77)
LMB	9.00 (3.41)	9.21 (3.64)	8.89 (3.80)	9.01 (3.97)	9.23 (3.59)	8.93 (3.18)
LM- A del	7.03 (3.95)	7.60 (4.30)	7.51 (4.24)	7.84 (4.33)	7.61 (4.32)	7.46 (4.33)
LM- B del	6.54 (3.82)	6.94 (4.04)	6.77 (3.93)	6.70 (4.68)	7.10 (4.39)	6.34 (3.79)

MMSE, Mini Mental State Examination; EF composite, Executive Functioning Composite consisted of TMT A and B, Trail Making A and B in seconds, SDMT, Symbol Digits Modalities, items correct in 90 s, Animals, Animals recall in 60 s, F Words, Letter F in 60 s; VEM composite, Verbal Memory Composite consisted of HVLT trials, Greek version of Hopkins Verbal Learning Test-Revised, trials 1–4, LM A&B, Logical Memory A&B, LM – A &B del.

### Relationship Between Age, Education, EF and VEM

Pearson correlation analyses were conducted to examine relationships between age, education, EF and VEM. Preliminary analyses were performed to ensure no violation of the assumptions of normality, linearity and homoscedasticity. Results indicated that there were significant moderate-strong, negative correlations (Bonferroni *α*/*k* = 0.01) between age, VEM and EF across the three time points, respectively. Additionally, education yielded in significant moderate-strong correlations with EF and VEM across the three time points. The association between education and VEM increased across time, whereas the association between education and EF decreased. Table 4 depicts the correlations.

**Table 4:** Correlations between age, education and cognitive domains.

S. No.		1	2	3	4	5	6	7	8
1	Age	1							
2	Education	−0.293^**^	1						
3	EF, TIME 1	−0.479^**^	0.565^**^	1					
4	EF, TIME 2	−0.336^**^	0.574^**^	0.776^**^	1				
5	EF, TIME 3	−0.422^**^	0.442^**^	0.645^**^	0.717^**^	1			
6	VEM, TIME 1	−0.484^**^	0.401^**^	0.606^**^	0.503^**^	0.455^**^	1		
7	VEM, TIME 2	−0.403^**^	0.412^**^	0.494^**^	0.506^**^	0.476^**^	0.776^**^	1	
8	VEM, TIME 3	−0.439^**^	0.530^**^	0.501^**^	0.452^**^	0.403^**^	0.721^**^	0.804^**^	1

** *p* < 0.01.

### Time, Sex, Age, Education and Executive Functioning

The second analysis examined the effect of Time on EF while sex was conducted as between-subject factors and age and education included as covariates. No violations from preliminary checks were found (normality; linearity; homogeneity of variances; reliable measurement of the covariates). Time as main effect did not yield significant changes on EF performance, *F*(2, 167) = 0.923, *p* = 0.39, partial *η*^2^ = 0.01, observed power = 0.2 indicating that participants exhibited no significant change on EF across the three testing periods. No main effect was found for sex, *F*(1,168) = 1.26, *p* = 0.26, partial *η*^2^ = 0.08, observed power = 0.23, on EF after partialing out the effect that the covariates has on the outcome. Age and Education significantly predicted the EF performance *F*(1, 168) = 11.23, *p* ≤ 0.05, partial *η*^2^ = 0.05, observed power = 0.91; *F*(1, 158) = 90.03, *p* < 0.001, partial *η*^2^ = 0.36, observed power = 1. There was a significant interaction effect between time and education, *F*(2, 167) = 7.02, *p* < 0.001, partial *η*^2^ = 0.08, observed power = 0.9. As observed on Table 4, the relationship between education and EF, while significant, it decreased from Time 1 to Time 3. None of the other multivariate interactions (time × age; time × sex) were significant. These finding indicate that while the overall performance on EF tasks does not change significantly across time, education does have a positive influence on the patterns of change. The patterns of change did not differ between males and females.

### Time, Age, Education, Sex and Verbal Episodic Memory

The third analysis examined the effect of Time on VEM. Sex was a between-subject factor and age and education were included as covariates. No violations from preliminary checks were found (normality; linearity; homogeneity of variances; reliable measurement of the covariates). Time as main effect again did not yield significant changes on VEM performance, *F*(2, 170) = 0.680, *p* = 0.84, partial *η*^2^ = 0.002, observed power = 0.07 indicating that participants exhibited no significant change on VEM across the three testing periods. The main effect for sex was not significant, *F*(1, 171) = 1.03, *p* = 0.31, partial *η*^2^ = 0.003, observed power = 0.16. Age and Education significantly predicted the VEM performance *F*(1, 171) = 17.22, *p* = *p* < 0.001, partial *η*^2^ = 0.09, observed power = 1; *F*(1, 171) = 61.25, *p* < 0.001, partial *η*^2^ = 0.26, observed power = 1. In contrast with EF, none of the multivariate interactions (time × age; time × sex; time × education) were significant. These finding indicate that VEM performance was similar across the time periods and across the two sexes. Furthermore, while age and education are significant predictors of VEM performance, the patterns of performance were not affected by age and years of education.

### Effects of APOE ε4 on MMSE, EF, VEM, Baseline Performance

Three Mann–Whitney test were conducted to compare the MMSE, VEM and EF performance for *APOE* ε4 allele carriers and non-carriers. There was no significant difference between the two groups on the MMSE indicated that performance was not different for *APOE* ε4 carries and *APOE* ε4 non-carries, *U* = 3,233, *p* = 0.25. The distribution of EF and VEM was the same for *APOE* ε4 carries and *APOE* ε4 non-carries *p* = 0.99 and *p* = 0.47, respectively.

## Discussion

The Neurocognitive study for the aging is the first longitudinal project on cognitive aging in Cyprus. It offers a unique opportunity to study modifiable and unmodifiable risk factors, including demographic, biological and genetic indices, in respect to brain health. Study participants were community dwellers living independently, who were born and raised in a small Mediterranean country with its unique geopolitical, social-cultural and genetic characteristics. As noted in previous publications stemming from NEUROAGE (Constantinidou et al., 2012a; Philippou et al., 2018), the project has been able to capture the last generation of Cypriots who have attended very little formal schooling. While the average education is 9 years, there was a wide variability among study participants, ranging from 2–20 years. It is stressed that this is the last generation of individuals with low education. Since, following the establishment of the Republic of Cyprus in 1960, public education has been free and mandatory through grade 9 or age 15. The present study is our first effort to examine the longitudinal performance of study participants and examine change at two additional time points over a 4–5 year period in three key cognitive domains: overall cognition, EF and VEM. The contribution of key demographic and genetic variables, namely sex, age, years of education, and *APOE* ε4 were also examined.

### Demographic Factors of Age and Education and Cognitive Change

Older adults in our NEUROAGE cohort maintained overall cognitive performance across the five-year period as measured by the MMSE, the most widely used screening measure for global cognition. Age and education were not significant predictors of performance on the MMSE which adds to its versatility as a universal screening tool by a variety of health care professionals. Age systematically predicted performance on the VEM and EF measures, but was not associated with different patterns of cognitive change across the follow up period. The present findings are in contrast with longitudinal studies supporting significant changes in VEM and EF (Treitz et al., 2007; Lundervold et al., 2014) and in agreement with (Van Dijk et al., 2008) who used a similar experimental design. The difference between our study and studies demonstrating different findings might be due to methodological factors. For example, the average age of our cohort was a bit older (73 years) as compared to the Lundervold et al. (61 years), which may contribute to differences in findings. In addition, different measurements of verbal learning may influence study results. In the present study, the VEM composite consisted of both verbal learning tasks (HVLT-R Greek Version) and story recall tasks in order to obtain a more comprehensive representation of VEM. Future research will continue to explore the stability of VEM as more testing waves are recruited in the NEUROAGE project.

Another important finding of the present study was the stability of the EF performance across time. Several studies have provided evidence on the effects of aging on various aspects of EF. The literature is fairly consistent that EF abilities diminish as a result of biological age. However, this is the first study demonstrating that in cognitively and neurologically healthy older adults, EF abilities do not change significantly over a five-year period, as opposed to individuals with MCI or other neurodegenerative conditions.

In current literature, there has been a growing debate on the exact contribution of education in cognitive aging. In cross sectional studies, older adults with higher education perform better on VEM and EF measures. Education, is also the primary proxy measure included when estimating cognitive reserve, the mind’s ability to implement strategies and compensate for the effects of aging in healthy individuals (Giogkaraki et al., 2013). However, the existing aging literature is not conclusive on the potential influence of education on the pattern of change across time in individuals without dementia. Research based on longitudinal data report inconsistent findings concerning the link between education and patterns of cognitive decline (e.g., Alley et al., 2007; Ritchie et al., 2016). The present study makes significant contributions towards characterizing the contribution of education in cognitive change across time.

The findings indicate that education systematically predicted EF and VEM performance across the five-year period. Of importance is the finding that while education was positively associated with VEM at each time point, and in fact increased the association at Time 3, education did not influence the pattern of change on the VEM composite score across time. In contrast, years of education influenced the pattern of change on the EF composite for our cohort; those with higher education had better performance. This significant interaction was observed despite the fact that the association between education and EF, diminished across time. Previous research with our cohort analyzing baseline performance (Constantinidou et al., 2012a; Giogkaraki et al., 2013; Constantinidou et al., 2015; Philippou et al., 2018) demonstrated that younger participants and those with higher levels of education performed better as compared to older participants and those with lower levels of education on VEM and EF measures. Furthermore, cognitive reserve moderated the effects of biological age on VEM and EF, however, the moderating effect was higher for EF at baseline (Giogkaraki et al., 2013). Similar to our findings, Aschwanden et al. (2019) in their longitudinal analysis demonstrated that the highly educated sample showed better performance on memory in five-years, but the cognitive performance was rather stable over time despite of education. The present findings advance our current understanding on the beneficial effects of education in maintaining higher levels of EF performance across time. Future research should continue to characterize the slope of change across time across the key domains of VEM and EF.

### Biological Factors of Sex and *APOE* ε4 and Cognitive Change

Previous research has provided mixed evidence on sex differences and potential interaction with cognitive performance in healthy aging (e.g., McCarrey et al., 2016; Zaninotto et al., 2018). In the present study, sex did not appear to have an effect on performance at baseline or to differentiate cognitive performance patterns over time. While the VEM scores of our male participants diminished slightly across time, and the scores of the female participants were stable, those differences were slight and not statistically significant. Both sexes showed a slight but not significant change on EF across the three time points. Our findings are in agreement with other longitudinal studies (De Frias et al., 2006; Karlsson et al., 2015). In those studies, both males and females demonstrated a similar pattern of change across time on episodic memory, verbal fluency, visuospatial functioning and logical reasoning measures. The present study does not support the notion of a potential sex advantage for women for general cognition, VEM or EF at baseline or across time when controlling for critical factors such as age and education.

Finally, the *APOE* ε4 allele has been implicated as a primary genetic risk factor in pre-clinical and late-onset dementia. The present findings did not yield differences in the baseline performance between carriers and non-carriers on the MMSE, VEM and EF composite. This is in contrast to other studies suggesting that e4 is associated with memory decline, EF and general cognitive decline (Wisdom et al., 2011; Reas et al., 2019). It is important to note that in the present study, we selected individuals with a global cognitive score as measured with the MMSE of 24 and higher who completed all three cycles of the assessment. In a previous study examining the NEUROAGE cohort at baseline, we included individuals with lower MMSE scores. Carriers performed significantly lower than demographically matched non-carriers on the MMSE (Marsitopoulos and Constantinidou, 2015). Similar patterns were reported by Batterham et al. (2013). In their study, ε4 carriers scored significantly lower in initial memory performance and demonstrated greater decline in processing speed and word recognition than ε2 and ε3 carriers. However, after excluding 125 participants with low global cognition scores, all genotype effects became nonsignificant. As previously indicated, the frequency of the *APOE* allele ε4 in the Greek Cypriot population is among the lowest in Europe. Perhaps different patterns will emerge as we analyze future waves of the NEUROAGE cohort.

### Clinical Implications, Limitations and Future Studies

This study contributes to the promising idea of health aging in which older adults can maintain good brain healthy and cognitive performance into older age (Livingston et al., 2017). The study implemented sensitive clinical/diagnostic tools to measure cognitive change at baseline and across the longitudinal assessment of our NEUROAGE cohort. Our findings characterize the role of important demographic, biological and genetic indicators in individuals who are cognitively healthy and how these indicators interact over time in general cognition, memory and EF. The findings make significant contributions to our understanding of anticipated changes within a five-year period and contribute to the growing evidence on the exact contribution of sex, age and education in cognitive performance. As future waves of testing are completed, and as more participants are included in the testing cycles that will increase study power, we will be able to determine if the patterns observed in the present study are stable across longer periods of time and to detect potential change points in performance.

The current cohort of an island population is characterized by a low frequency of *APOE* ε4. Therefore, the generalizability of the current findings is limited to cohorts with similar characteristics. The study included a fairly homogeneous sample focused on the Greek Cypriot population which is the majority population residing in the Republic of Cyprus. Future studies should explore other ethnic and cultural groups residing in Cyprus, including Armenians and Maronites. Furthermore, Turkish Cypriots should be included in future studies, once the Cyprus problem is resolved and populations currently residing in the occupied part of Cyprus would be accessible.

While the current study focused on individuals with intact global cognition, future studies should include individuals with lower MMSE scores in order to capture the full spectrum of cognitive abilities and investigate change across time. Every effort was made by our team to retain study participants and to follow them up across time. The MMSE is a gross cognitive tool, so it is possible that some individuals with a score of 24–25 might experience mild cognitive changes that were not captured by the MMSE, but should have been captured by the EF and VEF.

The results of the study can guide clinical intervention trials aiming to improve cognitive performance in older adults. While formal education is typically acquired in early life, it is a significant predictor of cognitive health in later life. Furthermore, the present findings indicate that education clearly contributed to baseline performance for key cognitive factors (e.g., memory and EF), and also impacted the rate of cognitive change in healthy adult individuals. Since education is a key proxy for cognitive reserve, the present findings support the need to develop theory driven programs designed to improve cognitive functioning and facilitate EF and VEM. Research with the Categorization Program (CP), an intense neurocognitive rehabilitation program resulted in improved performance after a ten-week treatment period in healthy older adults who experience neurocognitive changes associated with the normal aging process (Constantinidou, 2019). Furthermore, gains generalized into novel tasks and were maintained after 4 months post training. Currently, we are exploring the utility of the CP in patients at risk for cognitive decline. In addition, we are exploring the effects of group interventions focusing on problem solving, EF and memory strategies combined with psychosocial training and emotion regulation strategies with our NEUROAGE cohort.

The present findings may provide some guidance on the patterns of cognitive testing required for cognitively healthy older adults, while also safeguarding valuable resources which could be diverted towards testing older populations with greater needs. While the study findings support cognitive stability for at least 4 years for our cohort, individual characteristics should be taken into consideration based on the current findings and from existing research for providing personalized advice in clinical practice. For example, those with lower education, those with higher cardiometabolic risk (Philippou et al., 2018), those with hearing and vision loss (Maharani et al., 2018) and those with depression and/or subjective cognitive decline (Constantinidou et al., 2015; Dimitriadou et al., 2018) should be closely monitored and tested more frequently. Future studies should incorporate contextual testing in the form of an informant report and direct patient observation because both EF and VEM play an integral part in effective participation during daily life activities (Constantinidou et al., 2015; Demetriou and Constantinidou, 2018). In a recent publication from the same cohort, healthy older adults reported changes in memory performance prior to their informants, suggestive of intact self-awareness (Dimitriadou et al., 2018).

Finally, given the complexity of biological aging, lifestyle habits, general health, metabolic and more genetic indicators could be important factors to be studied longitudinally in the context of cognitive/brain health, as they may play a mediating role for those with lower education. We are systematically investigating variables that affect aging such as cognitive reserve, psychosocial risk factors, hearing loss and cardiometabolic factors in order to develop multidimensional, theoretical and statistically valid prognostic models for successful cognitive aging (Maharani et al., 2018; Philippou et al., 2018). Currently, we are collecting data in order to explore the contribution of cognitive, social, and physical engagement to genetic and health factors to determine their potential contribution to adult brain health.

## Data Availability Statement

The datasets generated during and/or analyzed during the current study are not publicly available, but certain data could be made available from the corresponding author on reasonable request.

## Ethics Statement

The studies involving human participants were reviewed and approved by National Bioethics Committee (EEBK/ΕΠ/2008/26). The patients/participants provided their written informed consent to participate in this study.

## Author Contributions

AC collected data, coordinated data collection, conducted the data analyses, and wrote the manuscript. FC is the principal investigator of the study. She conceptualized and designed the project, coordinated the data collection, analyzed data, and wrote the manuscript. MH conducted genetic data analyses and revised the manuscript. SP collected clinical data and revised the manuscript. All authors contributed to the article and approved the submitted version.

### Conflict of Interest

The authors declare that the research was conducted in the absence of any commercial or financial relationships that could be construed as a potential conflict of interest.

## Footnotes

^1^http://www.who.int/countries/cyp/en/
